# Red wine and component flavonoids inhibit UGT2B17 *in vitro*

**DOI:** 10.1186/1475-2891-11-67

**Published:** 2012-09-07

**Authors:** Carl Jenkinson, Andrea Petroczi, Declan P Naughton

**Affiliations:** 1 School of Life Sciences, Kingston University, Penrhyn Road, Kingston upon Thames, London, Surrey, KT1 2EE, UK

**Keywords:** Red wine, Flavonoids, Testosterone, UGT2B17, Glucuronidation

## Abstract

**Background:**

The metabolism and excretion of the anabolic steroid testosterone occurs by glucuronidation to the conjugate testosterone glucuronide which is then excreted in urine. Alterations in UGT glucuronidation enzyme activity could alter the rate of testosterone excretion and thus its bioavailability. The aim of this study is to investigate if red wine, a common dietary substance, has an inhibitory effect on UGT2B17.

**Methods:**

Testosterone glucuronidation was assayed using human UGT2B17 supersomes with quantification of unglucuronidated testosterone over time using HPLC with DAD detection. The selected red wine was analyzed using HPLC; and the inhibitory effects of the wine and phenolic components were tested independently in a screening assay. Further analyses were conducted for the strongest inhibitors at physiologically relevant concentrations. Control experiments were conducted to determine the effects of the ethanol on UGT2B17.

**Results:**

Over the concentration range of 2 to 8%, the red wine sample inhibited the glucuronidation of testosterone by up to 70% over 2 hours. The ethanol content had no significant effect. Three red wine phenolics, identified by HPLC analyses, also inhibited the enzyme by varying amounts in the order of quercetin (72%), caffeic acid (22%) and gallic acid (9%); using a ratio of phenolic:testosterone of 1:2.5. In contrast p-coumaric acid and chlorogenic acid had no effect on the UGT2B17. The most active phenolic was selected for a detailed study at physiologically relevant concentrations, and quercetin maintained inhibitory activity of 20% at 2 μM despite a ten-fold excess of testosterone.

**Conclusion:**

This study reports that in an *in vitro* supersome-based assay, the key steroid-metabolizing enzyme UGT2B17 is inhibited by a number of phenolic dietary substances and therefore may reduce the rate of testosterone glucuronidation *in vivo*. These results highlight the potential interactions of a number of common dietary compounds on testosterone metabolism. Considering the variety of foodstuffs that contain flavonoids, it is feasible that diet can elevate levels of circulating testosterone through reduction in urinary excretion. These results warrant further investigation and extension to a human trial to delineate the health implications.

## Introduction

Numerous reports have attested to the health damaging effects of red wine and its components beyond excess alcohol consumption, for example - owing to pesticide and heavy metal content [1,2]. In contrast, many reports point to the health protective effects of red wine owing to the abundance of anti-oxidants [3,4]. Beyond modulating oxidative damage, one focus has been on the female endocrine system, following the reports that red wine has anti-aromatase properties [5]. This discovery broadened the debate regarding the link between alcohol intake and risk of developing breast cancer [6,7].

Equally, the associations of high and low testosterone levels with the development of various forms of prostate cancer have been subjected to considerable debate [8-10]. Given the inhibitory effects of red wine on aromatase it is conceivable that red wine also affects aspects of testosterone metabolism. Although recent epidemiological studies have suggested red wine consumption is not a potential risk factor for prostate cancer [11,12], the effects of red wine on testosterone metabolism warrant investigation.

Glucuronidation is a major metabolic pathway for the elimination of testosterone and numerous compounds from the body [13,14]. Glucuronidation of testosterone involves the transfer of a glucuronosyl group from UDP-glucuronic acid (UDPGA) to form the steroid conjugate, testosterone glucuronide, which is then excreted in urine [13]. The UDP-glucuronosyltranferase UGT2B17 enzyme is the main steroid glucuronidation enzyme of the UGT isotopes with more than double the glucuronidation activity compared to the second most active enzyme involved in glucuronidation of testosterone, UGT2A1 [15].

The metabolism of testosterone by UGT2B17 has been shown to differ between individuals owing to the variations in the expression of UGT2B17, which has been found to alter with ethnicity, affecting the excreted steroid concentrations [16]. In *in vitro* studies, the rate of testosterone glucuronidation has also been shown to be reduced with inhibitors of UGT2B17, such as non-steroidal anti-inflammatory drugs [15]. Whilst various drugs and compounds are glucuronidated as a substrate and inhibit UGT2B17 [13], little is known about the inhibitory effects common dietary substances could have on UGT2B17 and testosterone glucuronidation.

Recently, green and white teas and purified catechin constituents have been shown to inhibit the key testosterone glucuronidation enzyme UGT2B17 in a supersome-based assay [17]. Red wine is another rich source of phenolic compounds that have been found to exert anti-oxidant health benefits in humans [18]. Given the inhibitory effects of green and white tea on UGT2B17, along with the debate on red wine and prostate cancer, it is timely to investigate if phenolic compounds in red wine have an inhibitory effect on testosterone metabolism and excretion.

The aim of this study was to analyze the inhibitory effects of a dietary red wine sample and the common phenolic compounds found in red wine, independent of the effects of alcohol, on the glucuronidation of testosterone through the inhibition of UGT2B17. A further aim was to study the potential inhibitory effect of the common wine by-product 4-ethylphenol on testosterone glucuronidation.

## Materials and methods

### Materials

Testosterone, acetonitrile, ethanol, gallic acid, chlorogenic acid, caffeic acid and quercetin were purchased from Sigma Aldrich (Poole, United Kingdom). Dimethyl sulfoxide, methanol and high performance liquid chromatography (HPLC) grade water were purchased from Fisher Scientific. The UGT2B17 enzymes where purchased as human UGT2B17 supersomes from BD Biosciences. UDPGA was purchased as a UGT reaction solution (mixture A) from BD Biosciences. The MgCl_2_ and Tris–HCl buffers, along with alamethicin were purchased together as a UGT reaction mixture (solution B) from BD Biosciences. The red wine sample used was a Cabernet-Syrah red wine purchased from a local supermarket (London). All solvents used where HPLC grade.

### Methods

For general screening, HPLC analysis of testosterone glucuronidation was conducted on an Agilent 1260 HPLC system using an Ascentis Supelco C18 column, 25 cm x 406 mm i.d., 5 μM at 25°C column temperature. The mobile phase was methanol and water (80:20) at a flow rate of 1 mL/min and a 100 μL injection volume. The remaining testosterone from the reactions was detected by UV detection at 246 nm using a diode array detection system. The results represent the SD of duplicate values.

To assay the effects of quercetin at low concentrations, an alternate highly sensitive HPLC method was adopted to analyze testosterone [19]. Testosterone was dissolved in acetonitrile and added as 1% v/v. The mobile phase was acetonitrile/water (39/61, v/v) at a flow rate of 1 mL/min. The injection volume was 50μL and detection at 245 nm. The results represent the SD of triplicate values.

The testosterone glucuronidation assay, described in the BD biosciences data sheet for the human UGT2B17 supersomes, employs a standard incubation mixture containing UDPGA (2 mM), alamethicin (25 μg/mL), magnesium chloride (8 mM) and pH 7.5 Tris–HCl buffer (50 mM) and deionised water comprising 50% of the overall reaction volume. Following incubation at 37°C for five minutes, the reaction was initiated by the addition of 0.2 mg/mL ice cold UGT2B17 supersomes. The reactions were stopped by the transfer of 100 μL aliquots to 100 μL ice cold acetonitrile, vortex-mixed with samples stored on wet ice. The samples were centrifuged at 10,000 x g for 5 minutes. The aliquots of the supernatants were then analyzed by HPLC.

In order to study the inhibitory effects of red wine, various volumes ranging from 2-8% of red wine were added to the reaction. The reactions were stopped after one or two hours and the remaining testosterone was analyzed to determine any increase in testosterone through the inhibition by red wine. The red wine sample had been evaporated to dry residue to remove the ethanol and reconstituted with the same volume of water and filtered by a Millex 0.45 μM filter device.

The phenolic compounds gallic acid, caffeic acid and quercetin that are present in red wine were analyzed for the inhibition of UGT2B17. The phenolic standards (250 μM) were dissolved in ethanol and heated and mixed to aid dissolving where necessary and added to the reaction as 1% v/v of the reaction. The phenolic compound 4-ethylphenol was dissolved in ethanol and added to the reaction at 1% of the overall reaction volume at 750 μM overall concentration. The reaction duration was for 1 hour.

The red wine sample used in the glucuronidation assays was analyzed to determine the phenolic compounds present in the wine. The wine sample for HPLC analysis was prepared by evaporating the sample to dryness with the remaining dry residue dissolved in water to restore the original volume of the sample. The sample was then filtered by a Millex 0.45 μM filter device and injected into the HPLC. Quantification was performed as previously described [20] on an Agilent 1260 HPLC using a Kromasil C18 column, 250 mm x 406 mm 5 μM with detection at 280 nm. The mobile phase consisted of 0.1% orthophosphoric acid in water (A) and methanol (B). The mobile phase gradient elusions were (80% A: 20% B) 0 minutes, (60% A: 40% B) 10 minutes, (50% A: 50% B) 20 minutes, (45% A: 55% B) 30 minutes, (35% A: 65% B) 50 minutes. The flow rate was 1 mL/min with a 10 μL injection volume. Phenolic standards were dissolved in water at a concentration of 1 mg/mL and individually injected into the HPLC under the same conditions with the retention times compared to that of the red wine sample to indentify each phenolic compound.

Statistical analyses were conducted using SPSS v19. The significance of reduction in glucuronidation activity was tested for using a directional one-sample *t*-test (H_a_: activity <100%) with the significance level set at 0.05. Mixed model ANOVA was used to test for statistically significant difference over time and between concentrations, including testing for interaction effect.

## Results

### Inhibitory effects of red wine on UGT2B17

**Figure 1:**
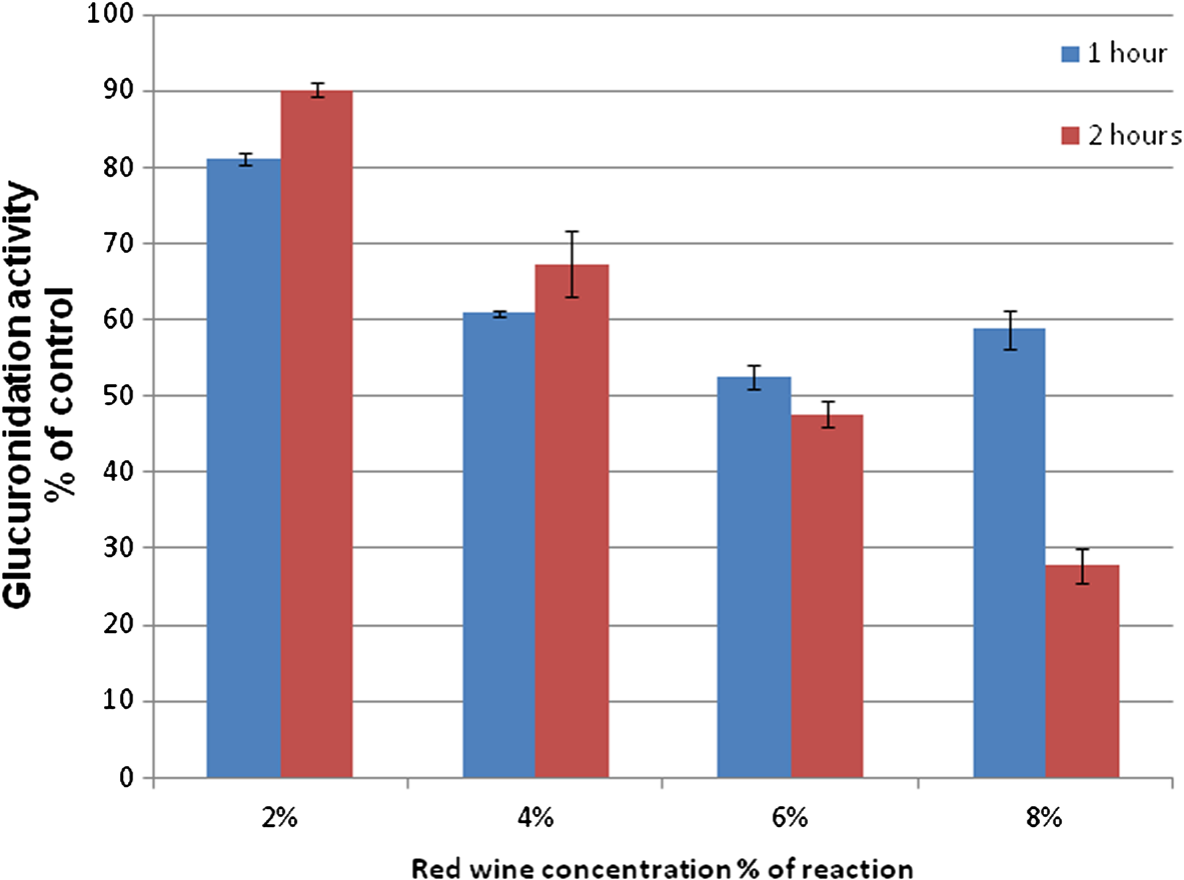
**Reduction of testosterone glucuronidation in the presence of red wine at 100 μM initial testosterone.** The results display the SD values of duplicate samples.

The effects of increasing concentrations of red wine on the UGT2B17-mediated glucuronidation of testosterone were assessed as a function of the reduction in conversion of testosterone to testosterone glucuronide (Figure 1). The results show that increasing the concentration of red wine resulted in a lower conversion of testosterone to its glucuronide conjugate. A reduction in UGT2B17 activity was observed for all, ranging between ca 10% to over 70% over two hours for additions of 2% to 8% red wine (p = 0.01; 0.002; 0.008 and 0.014 over one hour and p = 0.019; 0.033; 0.008 and 0.007 over two hours, respectively for 2%, 4%, 6% and 8%). The percentage of ethanol present in the final assay was in the range of 0.26% - 1.04% corresponding to additions of red wine at 2% - 8% respectively. It is notable that during a 2 hour period the inhibition is more pronounced at higher concentration of the red wine. Statistically significant differences were evidenced between times (p = 0.028) and concentrations (p < 0.001), with significant interaction between the two (p = 0.001).

The effect on UGT2B17 activity by the addition of an evaporated red wine sample reconstituted with an equal volume of water at concentrations of 4% and 8% of the reaction volume resulted in a glucuronidation % of control at 59.18 ± 3.154 and 23.48 ± 4.405, respectively. These values, taken after a two-hour duration, resemble those from the intact wine samples (Figure 1) indicating minimal contributions from the ethanol content on the inhibition of UGT2B17 by red wine.

**Figure 2:**
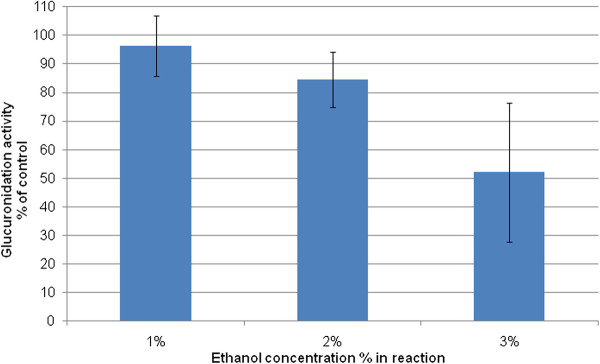
**Effect of ethanol on the testosterone glucuronidation by UGT2B17, based on the concentration of testosterone.** Concentration of testosterone was measured at the end of the reaction which was terminated after 30 minutes. The results display the SD values of duplicate samples.

The effects of increasing concentrations of ethanol on the reduction of testosterone by UGT2B17 are shown in Figure 2. The results indicated that testosterone glucuronidation was only slightly altered by ethanol at a 1% concentration (p = 0.353); however as the concentration of ethanol was increased to above 2% of the reaction volume, testosterone glucuronidation was affected as shown but not reaching a statistically significant level (p = 0.134 and 0.110 for 2% and 3%, respectively).

### Analysis of red wine

**Figure 3:**
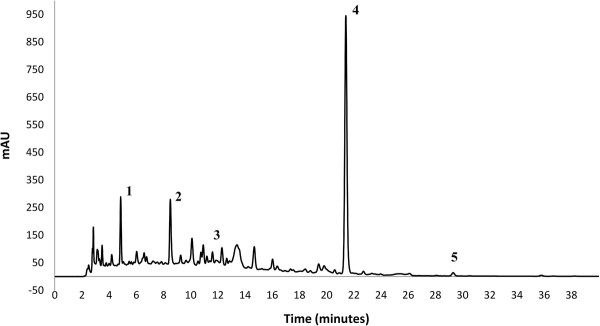
**Chromatogram showing the phenolic compounds present in the red wine sample.** 1. Gallic acid, 2. Chlorogenic acid, 3. Caffeic acid, 4. p-Coumaric acid, 5. Quercetin.

In order to identify individual inhibitors of UGT2B17, the phenolic content in the wine sample was investigated by HPLC. Analysis of the red wine confirmed the presence of gallic acid, chlorogenic acid, caffeic acid, p-coumaric acid and quercetin (Figure 3), which informed subsequent experiments.

### The inhibitory effect of individual phenolic compounds

Initial experiments were performed to screen the phenolics found in red wine for their effects on UGT2B17. Table 1 shows the effect on the glucuronidation activity of testosterone when 250 μM of each of the three phenolic compounds found in the red wine sample were added separately to the reaction over 60 minutes. Apart from chlorogenic and p-coumaric acids glucuronidation activity was reduced by each of the compounds in varying degrees. The inhibitory action of 4-ethylphenol is also displayed in Table 1. The results show the reduction in testosterone glucuronidation at initial testosterone concentrations of 100 μM, 50 μM and 20 μM.

**Table 1:** Reduction in testosterone glucuronidation activity of UGT2B17 by the addition of phenolic compounds

**Initial testosterone concentration (μM)**	**Test sample concentration**	**Test sample concentration (μM)**	**Glucuronidation % of control (±SD)**
*Screening study*			
100	4-Ethylphenol	750	78.9 ±1.131
50	4-Ethylphenol	750	57.3 ±13.548
100	Gallic acid	250	91.01 ±10.946
100	Caffeic acid	250	78.65 ±5.685
100	p-Coumaric Acid	250	NA
100	Chlorogenic Acid	250	NA
100	Quercetin	250	28.01 ±2.800
*Low concentration study*			
20	Quercetin	50	65.62 ±14.298
20	Quercetin	20	79.93 ±11.370
20	Quercetin	2	82.42 ±7.057
30	Quercetin	2	97.69 ±9.235
20	Quercetin	1.5	91.37 ±7.806

The results display the percentage change in the glucuronidation of testosterone over 60 mins by UGT2B17 in comparison to the uninhibited control.

Quercetin was selected from the initial high concentration screening assay for further study as it exhibited the highest level of inhibition at 72%. Reducing the testosterone levels to 20 μM resulted in inhibition of 34-18% by a low concentration of quercetin, in a concentration dependent manner, despite the 10-fold excess in testosterone levels (Table 1). In addition, for a quercetin concentration of 2 μM, increasing testosterone levels to 30 μM resulted in a reduction in inhibition from 18% to 2% suggesting that the mechanism is by competitive inhibition.

## Discussion

This report extends the previous study which demonstrated that tea and its component flavones competitively inhibit testosterone glucuronidation by UGT2B17. The results of this study showed that phenolic compounds commonly found in red wine, but also in many other foods, have comparable effect on testosterone glucuronidation. The rate of glucuronidation was similar on addition of the wine sample once the ethanol had been removed, indicating that it was likely to be the phenolic compounds that caused inhibition. Further studies revealed that ethanol had no effect at the concentrations found in the added wine sample. However, at higher concentrations of ethanol (>1%) the UGT2B17 enzyme activity was reduced. Ethanol has been linked to increased testosterone and aggression in male hamsters [21] and increased testosterone in rat brain [22]. From our results, the effects of alcohol on UGT2B17 are unlikely to account for the increase in testosterone, unless extremely high doses are consumed.

Several of the individual wine phenolic compounds inhibited the glucuronidation of testosterone at different efficiencies. The maximum inhibition was observed for quercetin, followed by 4-ethylphenol and caffeic acid. The serum concentrations of phenolic compounds that are commonly found in wine can be increased through supplementation such as with quercetin [23]. In one study, after supplementation, 1.5 μM quercetin levels in plasma were reported [24]. This concentration of quercetin affected a 9% reduction in UGT2B17 activity despite a high concentration of testosterone at 20 μM. The reported mean level of serum testosterone in adult males is 35.9 nM [25]. Given the inhibition is competitive; these much lower concentrations of testosterone should result in higher inhibition of UGT2B17 by quercetin. Future studies are warranted to investigate the effects of red wine and its components at physiological levels of testosterone.

In addition, plasma concentrations of caffeic acid have been shown to increase after the consumption of red wine [26]. Given these increases in serum concentrations through supplementation it may be possible to further reduce testosterone metabolism through glucuronidation.

The phenolic compound 4-ethylphenol has been found in red wines to produce distinct aromas [27], as well as being produced by the spoilage of yeast, hence it can be found in other dietary substances [28] and enters knowingly or unknowingly into the diet. 4-Ethylphenol has also been shown here to inhibit the glucuronidation of testosterone through the UGT2B17 enzyme. The inhibition of UGT2B17 by 4-Ethylphenol was found to be greater at the lower 50 μM level of testosterone. However, at high initial testosterone concentrations above 150 μM there was very little or no inhibition showing that increasing the concentration of testosterone will overcome the inhibiting compound. 4-Ethylphenol has been shown to be a substrate for UGT2B17 with a glucuronide being formed [13]; therefore it is likely that these compounds are acting competitively with the UGT2B17 enzyme.

Whilst it has been found that no correlation exists between the variations of the UGT2B17 genotype with alterations in circulating serum testosterone levels [29-31], another study has found significant differences in circulating testosterone with an increase in individuals expressing no copy number of UGT2B17 compared to individuals with one or two copies of UGT2B17 [32]. It has yet to be determined if any direct inhibition of steroid glucuronidation enzymes could alter the levels of circulating serum testosterone in addition to altering the levels of testosterone excreted in urine. These results augment the previous study revealing that tea catechins can inhibit testosterone metabolism by supersomes containing UGT2B17 [17]. The ubiquitous presence of quercetin and other active flavonols, along with the catechins, in many foodstuffs indicate that any *in vivo* effects may be common. These effects, if found to occur *in vivo*, may have a pronounced effect on people with endocrine disorders or very low levels of endogenous testosterone, owing to high levels of receptor expression to compensate. A further aspect, although not studied here, is the potential interaction of quercetin-containing foodstuffs with drug metabolism as some drugs are metabolized via the action of UGT2B17 [33].

In conclusion it has been found that a commonly consumed dietary substance, red wine along with phenolic compounds present in red wine, inhibit testosterone glucuronidation. These results have also shown that although some of these compounds are not substrates of UGT2B17 they can inhibit the enzyme in supersomes.

## Competing interests

The authors declare that they have no competing interests.

## Authors’ contributions

All authors contributed to the study design, data analyses and manuscript preparation. CJ completed the laboratory studies. All authors approved the final manuscript.
